# Presentation, Clinical Pathology Abnormalities, and Identification of Gastrointestinal Parasites in Camels (*Camelus bactrianus* and *Camelus dromedarius*) Presenting to Two North American Veterinary Teaching Hospitals. A Retrospective Study: 1980–2020

**DOI:** 10.3389/fvets.2021.651672

**Published:** 2021-03-22

**Authors:** Taylor R. Locklear, Ricardo Videla, Ryan M. Breuer, Pierre-Yves Mulon, Mary Passmore, Jonathon P. Mochel, Rick Gerhold, John J. Schaefer, Joe S. Smith

**Affiliations:** ^1^Department of Large Animal Clinical Sciences, College of Veterinary Medicine, University of Tennessee, Knoxville, Knoxville, TN, United States; ^2^Wisconsin Veterinary Diagnostic Laboratory, University of Wisconsin–Madison, Madison, WI, United States; ^3^Department of Medical Sciences, School of Veterinary Medicine, University of Wisconsin–Madison, Madison, WI, United States; ^4^Department of Biomedical Sciences, College of Veterinary Medicine, Iowa State University, Ames, IA, United States; ^5^Department of Biomedical and Diagnostic Sciences, College of Veterinary Medicine, University of Tennessee, Knoxville, Knoxville, TN, United States

**Keywords:** camels, trichostrongyle, trichuris, coccidia, capillaria, nematodirus

## Abstract

Old World Camelids (OWC) represent two species (*Camelus bactrianus* and *Camelus dromedarius*) with increasing numbers in North America. Gastrointestinal (GI) parasitism is a major cause of clinical disease in camelids and leads to significant economic impacts. Literature reporting on clinical parasitism of camels is localized to India, Africa, and the Middle East, with limited information available on OWCs in North America. Objectives of this study were to report on clinical presentation and diagnostic findings in *Camelus bactrianus* and *Camelus dromedarius* with GI parasitism and provide a comparative analysis between geographic regions. Medical records of OWCs presenting to two veterinary teaching hospitals (of the University of Tennessee and University of Wisconsin) were evaluated. Thirty-one camels including 11 Bactrians and six dromedaries (14 species not recorded) were included for the clinical component of this study, reporting on signalment, presenting complaint, and clinical pathology. Anorexia, weight loss, and diarrhea were the most common presenting complaint. Clinical pathology findings included eosinophilia, hypoproteinemia, and hyponatremia. For the second component of this study, a total of 77 fecal parasite examination results were evaluated for parasite identification and regional variation. *Trichuris, Capillaria, Strongyloides, Nematodirus, Dictyocaulus, Moniezia*, and protozoan parasites (*Eimeria, Cryptosporidium, Giardia*) were recorded. Strongyle-type eggs predominated, followed by *Trichuris* and *Eimeria* spp. There was a statistically significant variation in prevalence of coccidia between the two regions, with fecal examinations from Tennessee more likely to contain *Eimeria* (*P* = 0.0193). Clinicians treating camels in North America should recognize anorexia, weight loss, and diarrhea combined with clinical pathologic changes of hypoproteinemia, eosinophilia and hyponatremia as possible indications of GI parasitism. Clinicians should also consider the potential for regional variation to exist for GI parasites of camels in different areas of North America.

## Introduction

Old World Camelids (*Camelus bactrianus* and *Camelus dromedarius*) have growing popularity throughout the United States (US). In 2011, it was estimated that at least 2,000 dromedaries and 300–500 Bactrian camels were in the US (1). In the US, most camels are kept by zoos for entertainment or educational purposes with small numbers privately owned for work, breeding, exhibition, or as pets. Globally, camels are used for work as well as meat and milk production, or for racing (1–3).

Gastrointestinal (GI) parasitism is a significant cause of economic loss in domesticated ruminants (4) as well as pseudoruminant species, such as camelids. This is especially prevalent in geographic locations where warm temperatures and moisture are favorable to parasite transmission and survival in the environment. Parasitism is a common health concern for South American camelids in the US, causing increased morbidity and mortality with subsequent reduction in fiber quality, feed utilization, and fertility (5). Nematodes are commonly reported in South American camelids, including those that infect domestic ruminants in the US, such as *Ostertagia, Trichostrongylus*, and *Haemonchus* (5, 6). Camelid specific parasites have also been reported, such as the coccidian parasite *Eimeria macusanienesis* and nematodes, such as *Nematodirus lamae* (5, 6). A review of fecal samples in semi-captive guanacos found *Nematodirus, Marshallagia, Trichuris, Strongylida*, and *Eimeria* spp. (7). Parasitism in llamas and alpacas inhabiting North America is comparable to small ruminants, including *Haemonchus contortus, Trichuris* spp., and coccidia (7).

Currently there is a paucity of studies reporting clinical parasitism of OWCs in North America. The literature identifies coccidia, Cestodes, and gastrointestinal nematodes (such as *Strongyloides* and *Nematodirus* spp.) as common parasites in camels; however, this information is reported from predominantly Indian and Middle Eastern (ME) camels from Iran, Egypt, and Algeria (8–11). The objective of this study was to report the species of parasites found in camels presented to two US veterinary teaching hospitals, and to determine if there are regional differences in gastrointestinal parasites identified in fecal exam findings between the regions of the two veterinary hospitals. Additionally, another objective was to report clinical presentation and diagnostic test findings of those camels.

## Materials and Methods

This study had a retrospective design and was divided into two components: a clinical component, as well as a parasite identification and regional variation component.

### Clinical Component

For the clinical component, medical records of camels presented to the Veterinary Medical Center at the University of Tennessee College of Veterinary Medicine (UT) and the Morrie Waud Large Animal Hospital at the University of Wisconsin School of Veterinary Medicine (UW), between the dates of March 1980 and December 2020 were examined. Inclusion criteria were all cases for whom a diagnosis of internal parasitism was performed, regardless of the presenting complaint. Incomplete or partial medical records were excluded from the analysis. It was not required that the camel's presenting complaint or clinical signs be consistent with gastrointestinal parasitism. Camels presented to the hospital for whom no diagnosis of gastrointestinal parasitism was made were excluded from the study. Information collected from the medical records included species (dromedary, Bactrian, or unknown), age, gender, physical examination findings, hematology findings, parasitology exam results, and the diagnostic test results were reported when available. Repeat fecal examinations during the same visit were excluded. Hematology and clinical chemistry parameters were compared to multiple reported reference ranges for camel species (11–17).

### Parasite Frequency and Regional Variation Component

For determination of parasite frequency and regional variation of the observed parasites, fecal exam findings were compared from submissions to the UT Parasitology laboratory, and the Wisconsin Veterinary Diagnostic Laboratory (WVDL). Laboratory techniques utilized for both laboratories include modified Wisconsin fecal floatation, McMaster's fecal exam, and double centrifugation in a sugar solution with a specific gravity of 1.28. *Cryptosporidium* and *Giardia* diagnosis was performed with carbol-fuschin staining and fecal flotation, respectively. Due to the small number of observations, and mutual exclusivity of the specific observations, the composition of samples was compared with Fisher's Exact Test, with a *P*-value of <0.05 being statistically significant.

## Results

### Clinical Component

Of the cases that fit the inclusion criteria for the clinical component of this study, 29 camels presented to UT and two to UW, for a total of 31 camels presented to the veterinary hospitals. Sixty-eight records were excluded for lack of parasitism diagnoses or incomplete medical records. Of the camels identified in the clinical component, 48.4% (15/31) were female, 35.5% (11/31) were intact males, and 12.9% (4/31) were castrated males. The gender was not provided for one case (3.2%). Ages ranged from 3 months to 19 years of age, with the average age being (mean ± SD) 6.1 ± 4.4 years. The age of five camels was not defined, with one of these five having age non-specifically defined as being <1 year.

Of the cases presented for the clinical component of this study, the species was recorded for only 17 cases: 35.5% (11/31) were Bactrian camels (*Camelus bactrianus*) and 19.4% (6/31) were dromedaries (*Camelus dromedarius*). The species for the remaining 14 camels was not provided. Fourteen of the cases were from zoological facilities; for two cases, ownership was described from private individuals and ownership status was not provided for the remaining cases. Both cases from UW presented once. Multiple cases from UT (9/29) represented to the hospital more than once. Fecal exam results and presenting complaints for these camels were included in analysis as long as it was a new visit to the hospital.

#### Presenting Complaint and Clinical Signs

Camels presenting to the hospitals in this study most commonly presented for clinical signs consistent with parasitism, such as weight loss (*n* = 9 presenting complaints; *n* = 2 historical complaints), diarrhea (*n* = 9 presenting complaints; *n* = 3 historical complaints), or a reduction in appetite (*n* = 13; *n* = 8 inappetance; *n* = 2 hyporexia; *n* = 3 anorexia). With respect to reduction in appetite, eight cases presented for inappetance, two for hyporexia, and three for anorexia, with one additional case presented for abnormal behavior described as eating dirt. Three cases presented specifically for parasitism (described as “probable parasitism” or in one case, “whipworm infestation” in the record). Several cases also had a history of clinical signs that fit this category, although this was not necessarily the inciting cause for presentation to the hospital. Four cases presented for elective procedures (quarantine and pre-shipment exams, as a companion animal, and for castration). Table 1 displays all presenting complaint and historical information.

**Table 1:** Presenting complaints and historical clinical signs of camels with gastrointestinal parasites presented to two veterinary teaching hospitals.

**Clinical sign**	**Presenting complaint**	**Historical**
**Change in feed intake**		
Inappetance	8	
Hyporexia	2	
Anorexia	3	
Abnormal behavior (eating dirt)	1	
**Change in feces**		
Diarrhea	9	3
Soft feces	1	2
Blood in stool	1	
Weight loss	9	2
Lethargy	6	
Rectal prolapse	1	1
Abdominal signs		
Straining (urination or defecation)	2	
Colic	2	
Bloat	2	
Anemia	1	
Neurologic signs	2	1 (dead on arrival)
Recumbency (down)	2 (1 L5 fracture, 1 unknown etiology)	2 (dead on arrival)
Other trauma	1	1
Elective	4	

Weight loss (*n* = 9) and diarrhea (*n* = 9) were the second most common presenting complaint. Three cases were noted to have had prior history of diarrhea. One case presented for soft feces and two cases had a prior history of soft feces; one case presented for blood in the stool (hematochezia vs. melena was not clarified). One case, which presented for castration, developed diarrhea in hospital, which was attributed to stress and later, parasitism. Anemia was included as a presenting complaint for one case also presenting for weight loss. Six cases presented for lethargy.

Recumbency and colic were non-specific signs reported in the camels in this study. Two cases presented for recumbency, one of which was secondary to an fifth lumbar vertebral body fracture. Two cases presented as dead on arrival after a history of recumbency; in one case, neurologic signs preceded recumbency and death. Two additional cases had neurologic signs prompting presentation.

One camel presented for general colic signs; one presented for colic signs preceded by straining to urinate or defecate. One additional case presented for generalized straining to urinate or defecate and one case presented for a rectal prolapse. Rectal prolapse was noted in the recent historical information of one case and on physical exam in a second case. Finally, one case presented for trauma to the thorax and axilla. Two cases of subcutaneous edema at necropsy were reported. In one case it was clarified as thoracic and abdominal edema; this was a Bactrian camel with an immense strongyle-type burden (60,000 EPG).

#### Clinical Pathology

Twenty-six of the camels had complete blood counts and 28 camels had serum biochemistry analysis performed. Common clinical pathological findings from animals with clinical parasitism included eosinophilia (mean ± SD: 4.3 ± 4.4%), hypoproteinemia (mean ± SD: 5.6 ± 1.1 gm/dL), hypoalbuminemia (mean ± SD: 2.7 ± 0.9 gm/dL), and hyponatremia (mean ± SD: 145.5 ± 11.0 gm/dL). Complete clinical pathology findings are present in Table 2.

**Table 2:** Clinical pathologic findings of camels with gastrointestinal parasites presented to two veterinary teaching hospitals.

**Parameter**	**Unit**	***n***	**Mean ± SD**	**Minimum**	**Maximum**	**Reference range**
Packed cell volume/Hematocrit	%	25	27.3 ± 9.6	9.0	46.4	24–35 (17); 24–39 (12)
Red blood cell concentration	×10^6^/μL	24	8.1 ± 2.2	2.4	11.8	6.0–9.2 (17); 7.6–13.4 (12)
White blood cell concentration	×10^3^/μL	26	**17.4 ± 6.0**	**7.5**	**31.6**	11.0–16.0 (17); 2.9–16.5 (12)
% Segmented cells	%	25	73.9 ± 12.7	47.6	91.8	–
Segmented cells absolute	×10^3^/μL	24	12981.4 ± 5176.1	3,570	23992	–
% Band neutrophils	%	11	8.4 ± 8.3	1	30	–
Band neutrophils absolute	×10^3^/μL	10	2115.6 ± 2779.3	250	9480	–
% Lymphocytes	%	25	16.8 ± 11.8	3	44.9	41.0 (17); 18–62 (12)
Lymphocyte absolute	×10^3^/μL	24	2595.8 ± 1362.2	476	5570	–
% Monocytes	%	23	2.6 ± 1.8	0	7	4.0 (17); 0–4.0 (12)
Monocyte absolute	×10^3^/μL	22	451 ± 362.7	0	1414	–
% Eosinophils	%	16	**4.3 ± 4.4**	**0.2**	**15.0**	3.0 (17); 0–4 (12)
Eosinophil absolute	×10^3^/μL	16	729.9 ± 861.6	10	2610	–
Fibrinogen	mg/dL	15	247 ± 141	100	600	200–400 (12)
Total protein	gm/dL	28	**5.6 ± 1.1**	**3.6**	**7.7**	6.3–8.8 (17); 6.42 ± 0.4 (14)
Albumin	gm/dL	28	**2.7 ± 0.9**	**1**	**4.4**	3.0–4.4 (17); 2.5–4.5 (12)
Globulin	gm/dL	26	**2.9 ± 0.6**	**1.4**	**3.9**	2.9–4.5 (17);
Sodium	mEq/L	24	**145.5 ± 11.0**	**109**	**160**	149–158 (15, 16)
Potassium	mEq/L	24	3.9 ± 0.8	2	5.6	3.6–6.0 (17)
Chloride	mEq/L	24	108.8 ± 11.9	65	123	106–123 (17)

*Bold value indicates deviance from reported reference range*.

#### Parasite Identification

Seventy-five fecal examinations were performed; one case of *Trichuris* diagnosed on a biopsy specimen of the colon and one case of an *Anoplocephalidae* tapeworm found on necropsy were included for a total of 77 parasite diagnoses.

Nematodes were the most identified phylum. Strongyle*-*type parasites predominated, as they were identified on 83.12% (64/77) fecal examinations. This was followed by *Trichuris* 50.65% (39/77), *Capillaria* 7.79% (6/77), *Strongyloides* 2.60% (2/77), and *Nematodirus* 2.60% (2/77). *Dictyocaulus* spp. was diagnosed in two cases (2.60%, 2/77). *Eimeria* were the second most common parasite, with *Eimeria* reported in 44.16% of cases (34/77). Other protozoan parasites included *Cryptosporidium* and *Giardia*. *Cryptosporidium* was noted in one case (1.30%, 1/77) and *Giardia* in 1.30% (1/77). Cestodes were less common, with *Moniezia* identified on 7.79% (6/77). A necropsy sample described a tapeworm from *Anoplocephalidae*, however genus was not clearly defined (1.30%, 1/77). Table 3 identifies the case diagnoses of parasite species. Mixed infections with two or more parasite species were identified in 65.94% (50/77) of cases. Supplementary Table 1 compares parasite distribution amongst camel species, no statistically significant differences were noted.

**Table 3:** Parasite species diagnosed in camels and camel fecal samples with gastrointestinal parasitism from two veterinary parasitology laboratories in North America.

**Parasites**	**UT diagnoses** **(*n* = 43)**	**UW diagnoses** **(*n* = 34)**	**Total diagnoses** **(*n* = 77)**	**Percentage of total diagnoses** **(*n* = 77)**
**Nematodes**				
Trichostrongyle-type	35	29	64	83.12%
*Trichuris*	19 (one on biopsy)	20	39	50.65%
*Capillaria*	2	4	6	7.79%
*Strongyloides*	2	0	0	2.60%
*Dictyocaulus*	2	0	0	2.60%
*Nematodirus*	1	1	2	2.60%
**Protozoa**				
*Eimeria*	24	10	34	44.16%
*Giardia*	0	1	1	1.30%
*Cryptosporidium*	1	0	1	1.30%
**Anoplocephalidae (Cestodes)**				
*Moniezia*	3	3	6	7.79%
Genus not reported	1 (on necropsy)	0	1	1.30%

### Regional Variation

Forty-one fecal examinations from UT were included as well as one intestinal biopsy sample and one necropsy finding, resulting in a total of 43 samples. Thirty-four samples from WVDL were included for a total of 77 fecal parasite diagnoses in this study. Note that some of these samples would have overlapped with animals from the clinical component of this study.

Of the 41 fecal examinations reported by UT and the 34 reported by WVDL, statistically significant differences were noted in the populations of camels that had coccidia present (*P* = 0.0193) present. When coccidia was considered 58.54% of UT fecal exams observed coccidia compared to 28.57% for WVDL. The results of the regional comparisons is displayed in Table 4.

**Table 4:** Comparison of fecal examinations from samples presented to the University of Tennessee (UT) and the University of Wisconsin (UW).

	**Strongyle**	***Trichuris***	***Eimeria***	***Capillaria***	***Moniezia***	**Mixed**
UT	34/41 (82.9%)	18/41 (43.9%)	24/41 (58.54%)	2/41 (4.87%)	3/41 (7.32%)	30/41 (73.17%)
UW	29/34 (85.29%)	20/34 (58.82%)	10/34 (29.41%)	4/34 (11.76%)	3/34 (8.82%)	20/34 (58.82%)
*P*	1	0.2486	**0.0193**	0.4011	1	0.2242

*A P-value of <0.05 (bold) was considered statistically significant (P <0.05)*.

## Discussion

Herein we present a description of clinical signs, parasite genera observed, and clinical pathological changes of camels presented to referral teaching hospitals for gastrointestinal parasitism in the US. Nematodes are the most significant parasites of South American camelids and appear to be of similar concern in camels (13). In a review of camels in Sokoto, Nigeria, nematodes were identified in 80.56% of camels surveyed (11). Nematodes were of similar prevalence in this study. Nematodes, including strongyle-type (which normally encompasses *Haemonchus, Trichostrongylus*, and *Ostertagia*)*, Strongyloides, Trichuris, Nematodirus, Capillaria*, and *Dicytocaulus* were identified.

The age of camels infected by coccidia in this investigation was broad, ranging from 10 months of age to 19 years of age (average: 5.6 ± 4.58 years of age). Our results contrast from previously reported that coccidia infections are most common in younger camels (<5 years of age) (18). However, another study identified that apparently healthy older camels have a high infection rate with respect to clinical coccidiosis (19). The youngest camel identified with coccidia (10 months of age) presented for weight loss and hyporexia yet had a low parasite burden. In the case of a 2 year old camel presenting for diarrhea, the diarrhea was accredited to the coccidia in the medical record. Interestingly, the youngest camel identified in this study was not positive for coccidia on fecal floatation (*n* = 1, 3 months of age). As the majority of camels with coccidia in this study had a mixed parasite burden, it is difficult to ascertain which parasite was responsible for the presenting clinical signs. All of these species can cause weight loss, diarrhea, and/or changes in appetite. Further information is needed to determine if older camels in this geographic region are more susceptible to coccidiosis than described in their native regions.

The common clinical pathological findings of the clinical cases in our study include hypoproteinemia (camels in this study: 5.6 ± 1.1 gm/dL; reference range: 6.3–8.8) (17); hypoalbuminemia (camels in this study: 2.7 ± 0.9 gm/dL; reference range: 3.0–4.4) (17); and hypoglobulinemia (camels in this study: 2.9 ± 0.6 gm/dL; reference range: 2.9–4.5) (17). Hyponatremia was also observed (camels in this study: 145.5 ± 11.0 gm/dL; reference range: 149–158) (15, 16), although this may not be a consistent finding as there is much variation in the reference ranges described for serum sodium in camels (12). A relative eosinophilia (4.3 ± 4.4%; reference: 3%) (17) was also observed. Similar to the camels in our study, loss of plasma protein is a common finding of gastrointestinal parasitism in North American domestic ruminants (20). Similarly the solvent drag caused by diarrhea has also been shown to contribute to increased loss of minerals (20), such as sodium. It is important to consider that there are many reference ranges described for hematology and clinical chemistry values of camel species, and with this in mind the authors selected general reported reference ranges of camel species for the investigation of this study.

Strongyle-type eggs were the most diagnosed parasite, found in 83.40% (64/77) of fecal examinations. Speciation for strongyle-type parasites was not provided for species whose strongyle-type eggs are not morphologically distinct. *Haemonchus* and *Trichostrongylus* have been diagnosed in camels (10, 21). *Haemonchus contortus* is a parasite of key concern in the Southeastern United States due to suitable environmental conditions, and is known to cause heavy parasitism in llamas and alpacas in the region (22). A common diagnostic finding in ruminants and camelids in the region with high *Haemonchus* burdens is anemia (4, 23–25), and several anemic camels with egg types suggestive of *Haemonchus* were identified in this investigation. An additional case in this study was described as having type II ostertagiosis based on necropsy findings and a heavy burden of strongyle-type eggs on fecal examination, but speciation of worms was not provided. Further information is needed to determine the most common strongyle-type parasite in camels in this region, however prior reports in camelids from the region and hospital suggests a high index of suspicion for *Haemonchus* spp. (23, 25).

*Nematodirus* and *Dictyocaulus* were variable in prevalence compared to other regions. *Nematodirus* has a reported prevalence of 10.71% described in Uttar Pradesh, Madhya Pradesh, Rajasthan (UMR) and as low as 5.30% described in Algeria, similar to the 4.65% (2/43) found in this study (9, 21). *Dictyocaulus* had a 0.56% prevalence, respectively in a population of camels in Algeria, less common than the 4.65% (2/43) found in this study (8). *Strongyloides* by comparison was uncommon, as it was only reported in 2.60% (2/77) of fecal examinations. Prevalence of 9–11% has been reported in other regions, as high as 32.14% in UMR (9, 10, 21).

*Trichuris* spp. appeared to be far more common in this study, found in 50.65% of fecal examinations, compared to other regions reported in the literature. Prevalence of *Trichuris* has been reported as 7.41% in Sokoto, and 29% in Bangladesh, respectively (10, 11). Prevalence as low as 2.23% was reported in a large-scale review of camel fecal examinations in Algeria (9). The results of this study were comparable to analysis of migratory camel in UMR, where a 50% prevalence was reported (21).

Coccidian parasites had a higher prevalence in this study compared to that reported in the literature; however, there was less variety in the protozoal parasites identified. *Eimeria* was identified in 44.16% (34/77), compared to a 25% prevalence reported in UMR (21). *Giardia* was described in one sample from WVDL (1.30%, 1/77), but is sparsely reported in the literature (26). *Balantidium*, a coccidian parasite reported to have as much as 22% prevalence in camels in Bangladesh (10) was not identified in our study. Similarly, *Entamoeba* has also been reported (21), and was not identified in our study.

Cestodes were uncommon, with one species of tapeworm identified (*Moniezia*). Prevalence of *Moniezia* was higher than reported, with 7.79% (6/77) found in this study compared to the 4.23% reported in Algeria and 2% described in Bangladesh (9, 10). The one report of a tapeworm in the family *Anoplocephalidae* was based on necropsy findings of a 9-month-old Bactrian camel; genus was not clearly described but the findings in this study are most suggestive of *Moniezia*.

Trematodes were not identified in this study. Prior reports of parasitism in camels express concern regarding identification of potentially zoonotic organisms, such as *Fasciola hepatica* (10), *Crypotosporidium* and *Giardia* spp. (27, 28), but there is a paucity of information for these parasites. Other trematodes identified in camels include *Schistosoma* spp., which were not identified in this study (10). Trematodes have been reported in cattle in the southern eastern United States, including *Fasciola hepatic*a (29). Lack of trematode identification in this study may be due to limited sensitivity of fecal screening to detect trematodes, which typically require diagnosis by sedimentation rather than flotation (30), however, evidence of trematodes was not noted on any necropsy findings. Further information is needed to determine prevalence of trematodes in camels in this region. Differences in management practices may play a role in decreasing the prevalence of trematodes in camels in this study. A small enclosure environment with controlled water sources, more typical to a zoo, may present a lower risk of coming in contact with trematode vectors than open pasture and access to wet environments.

Coccidiosis can be a cause of morbidity and mortality in young ruminants, including camels (31–33). Infections most commonly occur in camels 6 months to 2 years of age, and are characterized by severe diarrhea, dehydration, and possible death (31). In a review of Old World Camels from multiple regions in Africa and Asia, *E. camelii, E. rajasthani*, and *E. dromedarii* were the most commonly reported species of coccidia of camels (31). Speciation of coccidia found in the camels in this study was generally not provided. In some cases, brief morphologic descriptions were given, such as the parasite appearing most like *E. camelii*. It is important to note that *E. camelii* and *E. macuanensis* cannot always be routinely morphologically distinguished with the latter which commonly infects South American camelids, co-grazing of OWC and South American Camelids (pseudoparastism) may complicate this distinction.

There is little information regarding *Giardia* infection in camels. A review of cases admitted to a veterinary teaching hospital in Saudi Arabia found seven cases of camels with *Giardia* infection (26). There is some concern about the possible zoonotic potential of *Giardia* in camels, however overall it appears that there are fewer reports in camels regarding *Giardia* infection than *Eimeria* (28).

Whipworm infestations are described as a significant cause of morbidity and mortality in camels. In a case report from the Seoul Zoo, a 13-year-old dromedary developed mucohemorrhagic typhlitis and abomasal ulcers. This animal expired and death was attributed to whipworm infestation (34). Two younger dromedaries 3–4 years of age at the Seoul Zoo developed anorexia and chronic diarrhea in association with whipworms (34). In this study, one case which was dead on arrival was identified as having whipworms with small intestinal and colonic enteritis.

Bouragba et al. found *Cryptosporidium* in 1.81% of camels, primarily 1–4 years of age (9). The one case of Cryptosporidium in this study occurred in a young camel (3 months of age), however this camel presented for recumbency rather than diarrhea typical to clinical *Cryptosporidium* infection. While *Cryptosporidium* is one of the most investigated parasites of camels (27), only one documented outbreak of zoonosis from camels is reported, which occurred in Iran (19). Fayer et al. described a case where a camel shed *Cryptosporidium* over a 4 year period, as such the zoonotic potential of *Cryptosporidium* in camels requires further investigation (27, 28, 35).

*Dictyocaulus* has been reported in Algeria with a low prevalence of 0.56% (9). *Dictyocaulus* was identified in (2/77) of cases in this study. Interestingly, one case in this study was noted to have severe lymphoid hyperplasia at necropsy, which was credited to a high lungworm (presumed *Dictyocaulus* spp.) burden.

There was a statistically significant difference in the prevalence of coccidia between UT and UW. UW camels were less likely to have coccidia infections than UT. This difference was not likely due to variation in technique, as both laboratories utilized a 1.28 dilution with double centrifugation. Wisconsin generally has colder environmental conditions than Tennessee, which likely influences favorable conditions for parasites in camels in Tennessee. Differences in husbandry may also have a significant impact on parasite exposure; detailed information regarding management of the camels in this study was not provided (such as a camel in a solitary setting vs. a large group). Further information is required to determine regional variations of camel parasitism within the United States.

Eosinophilia and hypoalbuminemia have been identified in camels with trypanosomosis (36), hematologic abnormalities that were also found in this study population. Trypanosomosis can be a cause of lethargy, reduction in appetite, and poor body condition, making it a reasonable differential for those clinical signs in an imported camel. In reviewing the records, importation status was not able to be determined from the recorded historical information. If the camel's origin was included in the history, it was in regards to purchase from another facility from within the United States, or a few examples of captive-born calves. Due to the considerable expense of importing a camel when captive-bred camels are relatively accessible in the United States, it is unlikely there are many imported camels in this study. This is a limitation in assessing if trypanosomosis was a contributing factor to the clinical signs and hematologic abnormalities found in the camels in this retrospective evaluation. Trypanosomes were not noted to have been observed on any of the complete blood counts in this study, for which inspection of a blood smear is standard practice. Visual inspection of a blood film in one method for identifying trypanosomes. Future studies of camels in North America could also investigate the impact on importation vs domestic breeding status on the effect of parasitism, as well as focusing on the prevalence of trypanosomosis. Clinicians should be aware of this differential in anemic camels with a history of importation as it has been easily spread to non-endemic areas of Europe, such as France and Spain (37).

In addition to the retrospective nature and small sample size, there were several limitations with this investigation. This study incorporated camels from a limited geographic location, the eastern and midwestern United States. Further investigation is warranted to determine if there is regional variation in parasite burden amongst camels in other regions of the United States. Differences in management, including deworming practices, may also be a contributing factor in parasitism identified in this study. Camels in the United States are typically housed in zoological facilities, petting zoos, or for private ownership as a pet. When group size was reported, group size was small with only 2–3 individuals housed together. Co-grazing with other species was noted for several cases in this study (including equids), but we were unable to discertain this for all camels from medical records. In a petting zoo setting, exposure to small ruminants, bovids, and/or equids is possible. This could have a strong influence on the parasite exposure for camels. Additional work could determine the severity of clinical signs based on fecal egg burden. This was an initial goal of this study, but due to some results being quantitative (modified-Wisconsin) and some being qualitative (fecal flotation) it was not possible to examine this relationship in our study.

Further investigation is required to determine anthelmintic efficacy in camels, as well as identify resistance in parasites of camels in North America. Deworming dosage for OWCs is typically extrapolated from data in llamas and alpacas when available. Pharmacokinetic/pharmacodynamic studies in camelids, however, are limited, and dosage decisions for camels in North America are likely being selected based upon data for small ruminants or even bovids. In Peru, parasitism of New World Camelids is estimated to cause millions of dollars in losses annually (13). Camels may have future implications for sustainable goods (meat and milk) due to desertification, and a sequela of global climate change (2). As camels continue to have rising popularity worldwide, further data may become imperative in controlling parasite resistance and making judicious drug selection decisions regarding treatment, such as a “Test and Treatment” strategy prior to movement (38).

In conclusion, camels with clinical evidence of parasitism in the US commonly present with inappetance, diarrhea, and weight loss. Strongyle-type eggs, *Trichuris*, and *Eimeria* represent the most common parasites observed on fecal examination. Clinical pathology changes present in this population of camels includes eosinophilia, hypoproteinemia, and hypoalbuminemia. Clinicians should consider these clinical signs, common parasites, and clinical pathology changes when diagnosing, evaluating, and treating camels for which gastrointestinal parasitism is a differential.

## Data Availability Statement

The original contributions presented in the study are included in the article/Supplementary Material, further inquiries can be directed to the corresponding author/s.

## Author Contributions

TL, RB, and JSm developed the initial study design. TL, RB, JSm, RV, P-YM, MP, RG, and JSc contributed to further refinement of the investigation and contributed to the data collection. TL, JSm, and JM contributed to data analysis. All authors contributed to manuscript construction.

## Conflict of Interest

The authors declare that the research was conducted in the absence of any commercial or financial relationships that could be construed as a potential conflict of interest.
