# Supplementary material for: Presentation, Clinical Pathology Abnormalities, and Identification of Gastrointestinal Parasites in Camels (Camelus bactrianus and Camelus dromedarius) Presenting to Two North American Veterinary Teaching Hospitals. A Retrospective Study: 1980–2020
Source: Front Vet Sci. 2021 Mar 22;8:651672. doi: 10.3389/fvets.2021.651672 (PMC8019911; doi:10.3389/fvets.2021.651672)
Supplement: Supplementary file 1 [file Table_1.docx]

Supplementary Material

**Supplementary Table 1.** Comparison of fecal examinations from samples from dromedaries versus Bactrian Camels. A P value of <0.05 was considered statistically significant (P *< 0.05*).

|  | Trichostrongyle-type | *Trichuris* | *Eimeria* | *Capillaria* | Anoplocephalidae | *Strongyloides* | *Dictyocaulus* |
| --- | --- | --- | --- | --- | --- | --- | --- |
| Dromedary | 5/6  (83.33%) | 2/6  (33.33%) | 5/6  (83.33%) | 0/6  (N/A) | 0/6  (N/A) | 1/6  (16.67%) | 0/6  (N/A) |
| Bactrian | 9/11  (81.82%) | 6/11  (54.55%) | 6/11  (54.55%) | 1/11  (9.09%) | 2/11  (18.18%) | 1/11  (9.09%) | 2/11  (18.18%) |
| *P* | 1 | 0.6199 | 0.3334 | 1 | 0.5147 | 1 | 0.5147 |
